# On the complex relationship between resilience and hair cortisol levels in adolescence despite parental physical abuse: a fourth wave of resilience research

**DOI:** 10.3389/fpsyt.2024.1345844

**Published:** 2024-04-02

**Authors:** Wassilis Kassis, Dilan Aksoy, Céline Anne Favre, Julia Arnold, Stefan Gaugler, Katharina Elisabeth Grafinger, Sibylle Artz, Doug Magnuson

**Affiliations:** ^1^ School of Education, University of Applied Sciences, Windisch, Switzerland; ^2^ School of Life Sciences, University of Applied Sciences, Muttenz, Switzerland; ^3^ School of Child and Youth Care, University of Victoria, Victoria, BC, Canada; ^4^ Educational Psychology and Leadership Studies, University of Victoria, Victoria, BC, Canada

**Keywords:** physical abuse, family, resilience, depression, dissociation, aggression, adolescence, hair cortisol concentration

## Abstract

**Introduction:**

To understand the family’s role in adolescents’ mental health development and the connection to neurodevelopmental disorders related to experienced parental physical abuse, we first explored resilience pathways longitudinally and secondly, connected the identified patterns to adolescents’ hair cortisol levels that are rooted in the hypothalamic–pituitary–adrenal axis as the main stress response system and connected brain structure alterations.

**Methods:**

We analyzed longitudinal online questionnaire data for three consecutive high school years (from seventh to ninth grade) and four survey waves from a representative sample of *n* = 1609 high school students in Switzerland on violence–resilience pathways. Furthermore, we collected students’ hair samples from a subsample of *n* = 229 at survey wave 4. About 30% of the participating adolescents had been physically abused by their parents. Out of the overall sample, we drew a subsample of adolescents with parental abuse experiences (survey wave 1 *n* = 509; survey wave 2 *n* = 506; survey wave 3 *n* = 561; survey wave 4 *n* = 560).

**Results:**

Despite the odds, about 20–30% of adolescents who have experienced parental physical abuse escaped the family violence cycle and can be called resilient. By applying a person-oriented analytical approach via latent class and transition analysis, we longitudinally identified and compared four distinct violence–resilience patterns. We identified violence resilience as a multidimensional latent construct, which includes hedonic and eudaimonic protective and risk indicators. Because resilience should not solely be operationalized based on the lack of psychopathology, our latent construct included both feeling good (hedonic indicators such as high levels of self-esteem and low levels of depression/anxiety and dissociation) and doing well (eudaimonic indicators such as high levels of self-determination and self-efficacy as well as low levels of aggression toward peers).

**Discussion:**

The present study confirmed that higher cortisol levels significantly relate to the comorbid pattern (internalizing and externalizing symptoms), and further confirmed the presence of lasting alterations in brain structures. In this way, we corroborated the insight that when studying the resilience pathways and trajectories of abused adolescents, biological markers such as hair cortisol significantly enhance and deepen the understanding of the longitudinal mechanisms of psychological markers (e.g., self-determination, self-esteem, self-efficacy) that are commonly applied in questionnaires.

## Introduction

Family should serve a dual role, not only as a mitigator of existing psychiatric disorders but also as a type of space that fosters adolescents’ mental health development (1). This underlines the crucial role that family plays in children’s upbringing (2) and applies specifically where adolescents’ neurodevelopmental disorders (NDD) are concerned. As Ansorge et al. (3) have deduced and as others have continued to explore (see for example 4), there are several genetic and environmental risk factors that are likely to affect an individual’s risk of depression by influencing brain maturation. This understanding prompts a broader definition of NDD, encompassing not only neurodevelopmental but also psychiatric disorders (5) such as aggression, depression, or dissociation (6, 7). Therefore, in our study, we adopted a broader understanding of NDD (6, 8). We focused on these challenges as also being rooted in the hypothalamic–pituitary–adrenal (HPA) axis as the main stress response system and connected brain structure alterations (9).

Although the family should play a dual-protective role, it is unfortunately sometimes a risk factor. The alarming rates of parental physical abuse vary slightly across different regions; nevertheless, they present a very similar picture: In Switzerland, this rate stands at 19% (10), within the European Union it ranges between 20% to 25% (10, 11), and in the United States, it reaches 28% (12, 13). Regrettably, adolescent victims of parental physical abuse often refrain from reporting these incidents to the police, resulting in a significant concern regarding underreporting among young individuals. Severe physical parental abuse is detrimental to adolescents and affects their emotional well-being (14, 15), personal development (16, 17), and social adjustment (18, 19). These findings attest that many adolescents globally must endure violence on a regular basis within their families, despite expecting a safe and nurturing environment. Threatening experiences such as physical abuse by parents impede adolescents’ well-being and development. Predominantly, symptoms such as depression, anxiety, dissociation, and interpersonal aggression toward peers serve as central and indicative outcomes (14, 18). Meta-analyses have revealed that exposure to violence at home is associated with mental health and behavioral issues during adolescence (20). While researchers contend that it is crucial to consider the prevalence of more severe forms of physical abuse (12, 13, 21), even a single instance of such abuse can lead to enduring effects on mental health (13, 22).

Studies have shown that severity and type of family maltreatment (23) as well as time of adversity (24, 25) play important roles in HPA axis regulation. Adam et al. (26) summarized the influence of stress on HPA axis (dis)regulation, including: maltreatment, bullying, low socioeconomic status, violence exposure, and discrimination. Note also that acute exposure to these stresses is often associated with stronger acute cortisol reactivity. Although the exact pathway from the stressor to the development of mental health symptoms is not clear, HPA axis regulation seems to play an important role (27). Hyper- or hypo-cortisolism is considered “problematic as chronically higher or lower cortisol secretions are presumed to induce long-lasting alterations in brain structures involved in depression and anxiety disorders such as the amygdala, the hippocampus, and the prefrontal cortex” (9, p. 21).

Surprisingly, internationally about 20–30% of the adolescents who are physically abused by their parents are not showing any negative symptoms and can be described as resilient (15). Resilience is a concept and process which involves multiple systems, and it encompasses shared responsibilities among individuals and societal systems, each with varying degrees of influence (28). Defining the criteria for resilience can pose a challenge because it encompasses multiple indicators, which must be selected according to a specific adversity, a domain (such as school) and a developmental phase (29). As studies with the first two survey-waves of our project have shown (30, 31), family violence experiences may lead not only to neurodevelopmental disorders such as depression and dissociation, but also to significantly higher cortisol levels (25, 32, 33), which can impair adolescents’ personal, social, and academic development (31). Based on our previous work on resilience pathways in adolescence despite family physical abuse (15), we expanded the data scope to four survey waves of data over three years and specifically related resilience pathways to HPA levels. To our knowledge, there is no empirical longitudinal data on these complex relationships in adolescence. Understanding and managing the multifaceted aspects of this issue is critical to the well-being and development of young people and for the implementation of effective support.

### Encompassing the resilience pathways for physically abused adolescents

We assert that, for abused adolescents to exhibit resilient trajectories when compared to other adolescents who are being physically abused, they should demonstrate excellence across various domains of adjustment, aligning with the perspective of Luthar et al. (34) who said that resilience was not a universal concept. There is no such thing as “resilience” in general; resilience always has to be specified in relation to certain developmental challenges (35). Based on this modern understanding of resilience development, it is essential to understand resilience processes according to Ungar & Theron (36) only in the context of atypical adversities, as each adversity is associated with different adaptation processes. Therefore, to identify resilience pathways of physically abused adolescents by their parents, we need to capture specific psychopathological and supportive indicators that match the developmental burden in focus (see 15, 37, 38).

As we have established in previous research (15, 39, 40), we need family abuse-specific resilience indicators that are not just focused on psychopathological symptoms for adolescent students who have endured parental physical abuse. To adopt this approach, we identified resilience as a multidimensional construct focused on the specific domain of adolescent students’ lives (15, 30, 31, 39). In order to incorporate a more comprehensive approach, we draw on key findings from research of Ryan & Deci (41) on well-being to examine resilience in the context of violence. We define violence resilience as a multifaceted concept that encompasses both emotional well-being and academic success. Therefore, indicators of violence resilience in adolescent students should encompass aspects of their current and future well-being, including their emotional, social, and academic performance, rather than just overall satisfaction or specific achievements.

Following Kassis et al. (15), when working with the first two survey waves of our study, we operationalized, to capture not only negative but also positive development, two central developmental dimensions of well-being in adolescence, namely the hedonic dimension (feeling good) and the eudaimonic dimension (doing well), as different but interrelated facets of latent resilience indicators despite family maltreatment, converted them from a general well-being approach to resilience pathways, and empirically validated them. It is important to understand that resilience processes vary based on the specific context, so interventions can be tailored accordingly. For example, what constitutes “functioning well” (eudaimonic dimension) in a family setting may differ from what is considered optimal in a school environment, as we have in our focus, highlighting the need for topological specificity in addressing resilience in different settings. This distinction is important to keep in mind when working with children and families in various professional roles.

Additionally, we considered indicators such as aggression, depression and dissociation that are associated with HPA axis regulation activity. The hedonic dimension refers to the presence of positive cognitive evaluative emotions, such as self-esteem, as well as the absence of negative effects, such as depression/anxiety and dissociation. The connection between self-esteem and the capacity to endure family abuse in adolescence is firmly established and recognizes that an individual’s overall self-value plays a vital role in resilience against violence during this phase (15). Adolescents possessing elevated self-esteem are less susceptible to feeling intimidated by academic obstacles because they evaluate their character according to other important traits, thus preserving a favorable self-perception and confirming their general sense of worth. As a result, self-esteem functions as both a consequence and a safeguard for adolescents in challenging circumstances, and it is a particularly crucial factor for those who have experienced parental physical abuse (30). Adolescent maltreatment is associated with mental health conditions such as depression and anxiety (42). Favre et al. (31) were able to show convincingly when working with the first two survey-waves of our study, that dissociative and depressive symptoms as a form of internalizing behavioral problems receive relatively limited attention in resilience research. However, dissociations as possible consequences of traumatic experiences are particularly associated with physical abuse and they represent one of the most significant deficits in self-integration by splitting off psychological functions (16, 43, 44). Thus, it is crucial to include the absence of dissociative symptoms when conceptualizing the hedonic dimension of violence resilience.

The eudaimonic dimension and school-specific environmental mastery involve positive social skills, self-efficacy, low levels of aggression toward peers, discipline-oriented behavior, and academic success at school, especially where the focus of resilience despite family abuse is concerned (15, 30, 31). Mastery at school is important for setting and achieving goals and for retaining control over one’s life, notably for young people who have experienced physical abuse from their parents. Self-efficacy is an essential component of the psychosocial characteristics required to successful mastery as a student because it plays a critical role in how individuals think, feel, motivate themselves, and act (45, 46). An increase in self-efficacy leads to a rise in well-being, and buffers against family abuse when compared to lower levels of self-efficacy (15). Furthermore, parental abuse strongly correlates with peer aggression (11, 15, 18, 30, 47, 48). Regarding the factors that cause problems in school adjustment and hinder achieving school performance in students, rather than focusing on self-efficacy and resilience, research has largely focused on the importance and role of discipline in the school environment (49, 50), such as the student’s capacity to remain attentive during class activities to achieve higher learning outcomes (51, 52). Being a successful student, and here for the first time we link academic success with socio-emotional development, also means achieving academic competence at school and not just feeling good at school (hedonic indicators). As well, mathematical competencies, especially in an age of information computer technology developments, are central for performance in school subjects and are extended to subjects such as languages (53, 54) or biology (55). Especially in countries with a high proportion of students with migration backgrounds, it is the better indicator for school success to ask for competence in mathematics than for language competence in the new country (56, 57).

### Hair cortisol concentrations in physically abused adolescents

Based on the first three waves of resilience research (19, 58), we have identified characteristics of children which matter for their resilience (first wave), run research on relationships and promotive interactions as potentially protective (second wave), and tested for resilience patterns through prevention and intervention programs (third wave). The goal of the fourth resilience research wave is to build a more complex multilevel analysis and to incorporate biology into social sciences (59), along with knowledge gained from the first three waves about fostering resilience pathways (60).

One of the hotspots, as Masten (61) calls it, for integration is the HPA axis as the main stress response system. HPA is the neuroendocrine link between perceived stress (a psychological marker) and cortisol as the physiological reaction (a biological marker) to stress which occurs when individuals encounter persistent or recurrent external threats to their psychological health (62), causing irregularities of cortisol release (63). Specifically, adolescents’ chronic physiologic stress can be measured with hair cortisol because the posterior vertex region of the scalp is an effective medium to determine cumulative cortisol levels, which is a valid, feasible, and reliable measure (62).

As it becomes feasible to measure biological markers such as genes or hormones, and to combine these biological markers with psychological markers gained by questionnaire data, we have to answer questions on adaptive functioning of complex systems differently because the roles of the respective individual students’ psychobiological systems interact with the psychobiological systems of other students and teachers in the classroom. These interactions influence adaptive patterns and the interplay of schools’ functioning with students’ development and they may be identified by complex statistical tools (64).

Concerning stressors, while acute stress is associated with increased cortisol levels, and studies regarding child maltreatment also suggested that this response was common (24), many studies also show an opposite response, suggesting the body seems to adjust to chronic stress (attenuation hypothesis; 65; hypoactivation hypothesis; 66). This may result in hyper- and hypocortisolism, when stress exceeds the individual´s ability to cope (allostatic overload; 67, 68). This is thought to be a protective function of the body, but again has further health consequences (25, 69). Chronic exposure is mostly associated with blunted cortisol reactivity, flatter diurnal cortisol slopes, and lower cortisol levels in adulthood, which indicate hypocortisolism, whereas academic stress seems to connect to hypercortisolism. Adam et al. (26) saw this difference in response as rooted in the threat to the social self. In their study, Doom et al. (27) distinguished between parental harshness (as threat) and parental disengagement (as deprivation), with threat having an increasing effect and deprivation a decreasing effect on overall cortisol output. Although Milam et al. (70) study showed no difference between varying stress measures, the negative association between hair cortisol concentrations (HCC) and dispositional optimism provided evidence that processing plays a role. Theory and some evidence suggest that both the body’s response to cortisol and the baseline levels of cortisol may shift from elevated to suppressed levels the longer a stressor lasts, and the more time has passed since its exposure (26).

A latent profile analysis of diurnal cortisol in adolescents with depressive symptoms revealed two profiles: elevated and suppressed cortisol rhythms (71). Other studies also suggest links between HCC and depression and anxiety symptoms, but the strength and direction of these links vary widely (9). Koumantarou Malisiova et al. (72) showed in their systematic review that patients with depression displayed a trend for hypercortisolism, while patients with posttraumatic stress disorder showed a trend toward hypocortisolism. Zajkowska et al. (73) described the possible pathways of hyperactivation as via the reduction of neurogenesis by inflammatory cytokines, which, for example, lead to a reduction of serotonin, or via the immune system, which can cause changes in the brain. As for general populations, although cortisol levels are generally associated with antisocial behavior including aggression, the strength varies by age and the cortisol measurement method (74). Some studies found no association between cortisol and aggression in adolescence (see meta-analysis by 75). In turn, Blankenstein et al. (74) meta-analysis reported that nine out of eleven studies showed an association between low basal cortisol and aggression, while three studies indicated that higher basal cortisol associated with increased aggression. In relation to peer victimization, Arbel et al. (76) observed a positive association with peer aggression for both males and females, along with the finding that in male adolescents, this association was bi-directional. Furthermore, Babarro et al. (77) found a positive association between experiencing concurrent peer victimization and aggression and higher HCC scores.

Cortisol and cortisone production takes place in the adrenal cortex and the HPA axis controls it. This axis reacts to stress by releasing higher quantities of stress hormones such as cortisol (78). During hair growth, cortisol and other steroids are incorporated into the hair shaft, and according to the free hormone hypothesis (79), only unbound, free hormone fractions should be incorporated into the hair (80). Using hair to quantify steroids in a test subject has the advantage that long-term monitoring is possible, sampling is noninvasive; hence, the bodily integrity is kept intact, storage and shipment of samples are easy, and only small samples are required for analysis (between 10–30 mg., depending on the analytes and analysis; 81).

On average, scalp hair grows 1cm per month; hence, quantifying steroids in samples that are 3cm in length represents the cumulative steroid concentrations over a three-month period. Because steroids are endogenous, their analyses are more complex, because no analyte-free matrix is available. Different methods are available to determine steroid concentrations in hair, each with their own advantages and disadvantages (82). In the present study the so-called surrogate method was applied. Using this approach, quantification of analytes is performed using an authentic matrix spiked with surrogate analyte, which is commonly a stable isotope-labeled form of the analyte (82). This method provides reliable results both for humans and for mammals (82–86).

### Longitudinal insights on violence-resilience

While identifying the hedonic and eudemonic patterns of adolescent students at a specific time point represents an important initial step for understanding violence-resilience pathways in adolescence, gaining an understanding of how these patterns evolve over time is crucial for designing targeted school prevention and intervention programs. However, currently, there is a lack of data addressing how these patterns change over time, as well, the dynamics of these patterns during adolescence remain unclear. Although there is evidence indicating both stability and instability on the respective resilience patterns, there is a greater body of evidence supporting the notion of instability, suggesting significant changes throughout adolescence (15, 87, 88). Additionally, when connecting violence-resilience indicators with NDD (24, 25), we must pose questions about psychiatric symptoms such as aggression, depression, or dissociation being connected to HPA (7) and examine this connection as an indicator of brain structure alterations (9), because physical abuse in adolescence may affect especially the amygdala, the hippocampus, and the prefrontal cortex (9).

Establishing the resilience pathways in adolescence despite family physical abuse longitudinally is of paramount importance in enhancing our nderstanding and definition of this phenomenon. While we currently lack information regarding the persistence of violence-resilience outcomes that encompass both hedonic and eudaimonic aspects, it is essential to explore what transpires after initially detecting resilience at the respective starting time point. After both the special conceptual significance and the empirical relevance of the specific hedonic and eudaimonic latent factors on these paths were empirically validated with two waves of data collection, it is crucial to investigate whether the resilience patterns identified at the beginning led to different resilience trajectories after three years and how these pathways are connected to the HPA axis. Resilience research has examined sociodemographic factors, such as gender, migration background, and socioeconomic status, but their effects on resilience outcomes have yielded inconclusive results (38–40).

To address this gap, we conducted our study with four survey waves to delineate the resilience pathways and we employed person-oriented methodologies such as latent class analysis and latent transition analysis. As Masten (28) and Luthar et al. (34) suggest, we need to look at the specific at-risk population, rather than the general sample, to identify appropriate resilience pathways. By focusing on subsamples that actually, not just potentially, face adversity, we have the opportunity to identify pathways to successful development despite the specific adversity. Additionally, and in previous research with the first two waves of our survey, we were able to demonstrate that the subsample of abused adolescents cannot be directly compared to adolescents without these experiences due to a lack of measurement invariance (30, 31, 89, 90). This approach allowed us to categorize individuals into distinct classes based on violence-resilience indicators and to estimate the likelihood of an individual belonging to a particular class at two time points. This enabled us to gain insights into the continuity of violence-resilience levels.

We achieved this by employing latent transition analysis (LTA), a longitudinal analytical technique designed to characterize transitions over time (91). Through this person-oriented approach, we aimed to estimate and gain insight into the continuity of navigation and negotiation levels among students at two time points. We conducted an LTA for all four single study waves to determine the direction of the transitions of the respective subjects and to see if these transitions were developmentally forward with higher levels of hedonic and eudemonic indicators or backward transitioning to lower levels of hedonic and eudemonic levels. This person-oriented method (92) enabled us to classify subjects into distinct classes.

### Present study

Our research team embarked on this study to investigate the effectiveness of hedonic and eudemonic indicators in predicting adolescents’ development over three years.

The results and findings from the first two waves of the survey (see 15, 30) formed an initial empirical and conceptual framework, which had to be further elaborated theoretically and empirically validated by additional waves of the survey. In addition, we wanted to examine the relationship between the resilience patterns to be identified and the cortisol levels of the respective adolescents. We therefore tested five exploratory hypotheses over a period of three years We focused on resilience patterns consisting of hedonic and eudaimonic indicators of adolescent students who have experienced parental physical abuse, and it examined the relationship between these and biological markers, a methodology not previously employed. These patterns were used to predict a biological marker, namely HCC levels, at the last measurement point.

We tried to achieve this by first conducting a cross-sectional latent class analysis (LCA) and second, a LTA, a longitudinal person-centered technique designed to characterize latent transitions over time (91). Resilience research, although rarely (90), has examined sociodemographic factors such as gender, migration background, and socioeconomic status, but their effects on resilience outcomes have yielded inconclusive results. Here, we conceive violence resilience as positive well-being and functioning within the school environment, incorporating both hedonic and eudaimonic indicators while controlling for sociodemographic variables.

Additionally, for a subsample, we tested the potential impact of the pattern membership at study wave 4 on the hair cortisol concentrations (HCC levels). Given the novelty of this approach, we formulated and tested five exploratory hypotheses to obtain more definitive insights.

*First*, we hypothesized that by introducing hedonic and eudaimonic indicators, we would distinguish distinct patterns of violence-resilience outcomes among adolescents who had experienced parental physical abuse at least once in their lifetime. Our initial expectation was to identify four resilience outcome patterns across all four time points (study waves), building on the first two study waves (15, 30).

*Second*, drawing from well-established research in the field of family abuse with the first two study’s waves 1 and 2, we anticipated identifying four cross-sectional patterns: resilience, externalizing, internalizing, and comorbidity (15, 30).

*Third*, by recognizing that resilience is a dynamic state rather than an unchanging trait, we expected to observe shifts in the identified resilience outcome categories at different time points (survey waves). According to the previously published results of survey waves 1 and 2 (e.g., 15, 30, 31), we predicted that the resilient and comorbid patterns would exhibit greater stability, while the externalizing and internalizing patterns would display a greater propensity for change.

*Fourth*, we hypothesized that pattern membership differs in terms of sociodemographic characteristics such as gender, migration background, and socioeconomic level (15, 30, 31).

*Fifth*, we examined the potential impact of the pattern membership at study wave 4 on the HCC levels of the adolescents in question. We expected that non-resilient adolescents would have higher HCC levels compared to resilient adolescents.

## Materials and methods

### Study and sample

The data were derived from a longitudinal study with four study waves that investigated how adolescents develop resilience when experiencing parental physical abuse. We conducted this study during early autumn 2020 (*M* age study wave 1 = 11.76 [*SD* age study wave 1 = 0.64]), early summer 2021 (*M* age study wave 2 = 12.28 [*SD* age study wave 2 = 0.56]), early summer 2022 (*M* age study wave 3 = 13.71 [*SD* age study wave 3 = 0.54]), and early summer 2023 (*M* age study wave 4 = 14.26 [*SD* age study wave 4 = 0.54]). The study enrolled entire seventh-grade classes of high school students from the German-speaking region of Switzerland. The necessary consent forms were acquired from both the students and their caregivers, and no extrinsic motivations were provided. The research project received authorization by the Ethics Committee of the School of Education, University of Applied Sciences and Arts Northwestern Switzerland. The research team delivered a brief verbal introduction about the online survey in each of the 142 classes across 44 high schools enrolled in this study. Subsequently, the students completed the questionnaire, a process that typically took about 60 min.

We ran t-tests between the four survey-waves (see **Table 1**) to analyze for mean differences on gender, migration background, and socioeconomic status between the overall samples at each study wave, worked with a longitudinal sample of *N* = 1609. For the three sociodemographic variables, we identified only small effects (all displayed Cohen’s d are far lower than <.4) between the overall samples and the respective “abuse” subsamples for four study waves. There were significantly higher percentages of adolescents with a migration background and a lower socioeconomic class in the “abuse” subsamples than the overall samples for all four study waves.

**Table 1:** Sample mean (and standard deviations) for all four waves of socio-demographic variables between the overall samples and the sub-samples of adolescences having experienced physical parental abuse.

	Wave 1			Wave 2			Wave 3			Wave 4		
	Overall Sample *n* = 1858	Sub-sample *n* = 560	*d*	Overall Sample *n* = 1764	Sub-sample *n* = 523	*d*	Overall Sample *n* = 1534	Sub-sample *n* = 560	*d*	Overall Sample *n* = 1620	Sub-sample *n* = 477	*d*
Gender % Female	1.50 (.50) 49.2	1.56* (.50) 45.4	-.11	1.50 (.50) 47.7	1.53 (.50) 48.9	–	1.52 (.50) 48	1.52 (.50) 48.4	–	1.53 (.50) 47.4	1.50 (.50) 50.4	–
Migration Background	.32 (.47)	.44*** (.50)	-.25	.30 (.46)	.44*** (.50)	-.30	.54 (.49)	.67*** (.47)	-.25	.56 (.49)	.65*** (.48)	-.18
% Migration Background	53.2	56.2		51.10	55.6		54.8	67.1		56.2	64.8	
Socio-economic status	2.11 (.55)	2.00*** (.56)	.20	2.13 (.57)	1.98*** (.59)	.25	1.95 (.63)	1.86** (.64)	.15	1.95 (.63)	1.83*** (.65)	.20
Low (%)	21.0	25.9		23.7	28.2		22.5	28.4		22.0	31.2	
Middle (%)	60.8	59.2		58.8	58.0		59.3	57.3		60.1	54.5	
High (%)	18.2	14.8		17.6	13.8		18.2	14.3		17.9	14.3	

*p <.05; **p <.01; ***p <.001. Cohen’s d is only reported when results are significant.

The attrition of the “abuse” subsamples from study wave 1 (*n* = 560) to study wave 2 (*n* = 523) of only 6.61% is very low. Between study wave 1 and study wave 2 participants, no significant differences existed regarding the tested sociodemographic variables (gender: *t*(560) = .904, *p* >.05.; migration background: *t*(560) = −1.483, *p* >.05.; socioeconomic status: *t*(560) = −.859, *p* >.05).

This also holds true for the comparison between study waves 2 and 3 as well for study waves 3 and 4: Due to this, we consider the abuse samples comparable to the overall sample.

### Measures

#### Exposure to *parental physical abuse*

The single-item indicator measured the prevalence of parental physical abuse, with participants reporting whether they had experienced abuse at least once in their lifetime. Responses were dichotomized into either (0) no or (1) yes.

#### Three hedonic indicators

The three hedonic indicators included self-esteem, symptoms of anxiety and depression, and dissociation. Self-esteem was assessed using the Rosenberg Self-Esteem Scale (2015), which included a short scale of five items rated on a four-point Likert scale (Cα_study wave 1 = .90; Cα_study wave 2 = .92; Cα_study wave 3 = .93; Cα_study wave 4 = .92). The higher the score, the higher the self-esteem, with participants answering questions such as, “In total, I am confident in myself.” The responses were median split (MED_study wave 1 = 3.00; MED_study wave 2 = 3.00; MED_study wave 3 = 2.80; MED_study wave 4 = 3.00) and dichotomized into either (0) low levels or (1) high levels of self-esteem.

Symptoms of anxiety and depression were measured using a modified version of the Hopkins Symptom Checklist (93), consisting of 24 items rated on a four-point Likert scale (Cα_study wave 1 = .96; Cα_study wave 2 = .96; Cα_study wave 3 = .96; Cα_study wave 4 = .97), from 1 to 4: “Not at all,” “A little,” “Quite a bit,” and “Extremely.” The checklist provides two scores: a total score and an additional score measuring depression. We used the total score. One item, on sexuality, was omitted from the original 25-item version due to the participants’ young age. Participants responded to statements such as, “I feel fear” and “Thoughts of ending my life.” The responses were then median split (MED_study wave 1 = 1.62; MED_study wave 3 = 1.46; MED_study wave 4 = 1.65) and dichotomized into either (0) low levels or (1) high levels of symptoms of anxiety and depression.

Dissociation was measured using a four-item short scale called the Dissociation Tension Scale Acute (94), which included items measuring depersonalization, somatoform, derealization, and analgesia. Participants rated these items on a four-point Likert scale (Cα_study wave 1 = .80; Cα_study wave 2 = .85; Cα_study wave 2 = .86; Cα_study wave 4 = .87), from 0 (not at all) to 4 (extreme). The responses were median split (MED_study wave 1 = 1.00; MED_study wave 2 = 1.00; MED_study wave 3 = 1.00; MED_study wave 4 = 1.00) and dichotomized into either (0) lower levels or (1) higher levels of symptoms of dissociation.

#### Four eudaimonic indicators

The study measured four eudaimonic indicators, which included self-efficacy, aggression toward peers, disciplinary problems at school, and mathematics grade.

Self-efficacy was assessed using the General Self-Efficacy Scale, developed by Jerusalem and Schwarzer (46), which measures optimistic self-belief in an individual’s ability to cope with life challenges. Participants responded to the six-item short scale on a 4-point Likert scale, with a range of 1 for “not true” to 4 for “completely true” (Cα_study wave 1 = .88; Cα_study wave 2 = .90; Cα_study wave 3 = .92; Cα_study wave 4 =.92). To categorize the responses for the LCA/LTA, we performed a median split (MED_study wave 1 = 2.83; MED_study wave 2 = 2.83; MED_study wave 3 = 2.50; MED_study wave 4 = 3.00) and dichotomized them into either (0) lower levels or (1) higher levels of self-efficacy.

To assess aggression toward peers, we used the German Self-Report Behavior Aggression-Opposition Scale (95), which consists of nine items measuring overt and covert aggression toward classmates. Participants responded on a four-point Likert scale, ranging from 1 for “never happened” to 4 for “more than once per week” (items were reversed scored for higher scores indicating higher levels on aggression against peers; Cα_study wave 1 = .83; Cα_study wave 2 = .84; Cα_study wave 3 = 87; Cα_study wave 4 = .89). Responses were categorized for the LCA/LTA using a median split (MED_study wave 1 = 1.2; MED_study wave 2 = 1.44; MED_study wave 3 = 1.20; MED_study wave 4 = 1.44) and dichotomized into either (0) low levels or (1) high levels of aggression toward peers.

Disciplinary problems at school were assessed using the German Self-Report Behavior Aggression-Opposition Scale (95) and consisted of eight items measuring undisciplined behavior during class such as throwing items around or shouting during class time. Participants responded on a four-point Likert scale, ranging from 1 for “never happened” to 4 for “more than once per week” (items were reversed scored for higher scores indicating higher levels on disciplinary problems; α_study wave 1 = .80; α_study wave 2 = .84; α_study wave 3 = .86; α_study wave 4 = .87). Responses were categorized for the LCA/LTA using a median split (MED_study wave 1 = 1.2; MED_study wave 2 = 1.44; MED_study wave 3 = 1.62; MED_study wave 4 = 2.00) and dichotomized into either (0) lower levels or (1) higher levels of undisciplined behavior at school.

Grade in mathematics: We asked the students what their latest report card grade was in mathematics, ranging from 1 (the lowest) to 6 (the highest). MED_study wave 1 = 4.00; MED_study wave 2 = 4.00; MED_study wave 3 = 4.00; MED_study wave 4 = 4.00.

#### Hair cor*tisol concentration*

The concentrations of the steroids of interest, including HCC, were determined using a validated high-performance liquid chromatography coupled to tandem mass spectrometry method (LC-MS/MS) (96). Hair samples were washed with ultrapure water followed by acetone, a 3-cm segment of hair was cut off, cut into small pieces, and was approximately 20 mg were exactly weighed in duplicates. Extraction was performed for two hours at 55°C in an ultra-sonic water bath using methanol (internal standards were added prior). The supernatant of this extract was dried followed by liquid–liquid extraction with ethyl acetate and water for one hour at −20°C. The organic supernatant was once again dried and reconstituted in the mobile phase.

We conducted quantification using an eight-point calibration curve. Because the analytes of interest were endogenous, their deuterated, or if available, ^13^C_3_ labeled reference standards, were used, which has been previously shown to deliver sufficient and adequate results (82). For cortisol, corticosterone, and cortisone, the calibration range was from 1.9 to 225 pg/mg, and for all other analytes, it was from 1.0 to 120 pg/mg analyte concentration in the hair. Each hair sample was measured in duplicates and the results were expressed as their arithmetic mean.

#### Covariates

Students’ gender was obtained from the respective class list, which we received from the class teachers. We assigned 0 for boys and 1 for girls. We used socioeconomic status (SES) as a proxy for the students’ socioeconomic background, which was determined by combining four indicators (ranging from 1 for the lowest to 3 for the highest SES; Cα = .71). Further, we asked the students about their mother’s and father’s highest level of school education (ranging from 1 for primary school/junior high school to 3 for university degree/higher education) and we asked them for an estimate of the number of books the family and the student owned (ranging from 1 for 0–5 books to 3 for 31 or more books).

Finally, for *migration background (MB)*, students who were born in Switzerland and had only a Swiss passport were considered not to have a migration background. Students who did not meet these conditions were classified as having a migration background (0 for no MB and 1 for with MB).

### Analytic strategy

Following the five hypotheses we first aimed to test the proposed conceptualization of violence-resilience outcomes, encompassing both hedonic and eudaimonic aspects and to identify patterns of these outcomes. Second, we investigated how these patterns changed over four study waves. Third, we aimed to determine whether the categorization method using LCA/LTA reduced the beta error of misclassifying nonresilient adolescents as resilient. Fourth, we tested if pattern membership differed in terms of sociodemographic characteristics. Last, we tested the effect of pattern membership on HCC levels of the respective students.

For the first goal, we compared the seven indicators (self-esteem, depression/anxiety, dissociation, self-efficacy, aggression toward peers, disciplinary problems, and grade in mathematics) using paired t-tests between the four study waves. To identify cross-sectional resilience outcome patterns separately for study waves 1 to 4, we applied LCA using the seven classification variables in adolescents with experience of physical abuse. Third, we conducted longitudinal LTA to examine any significant differences in the longitudinal classification variables for the resilience-outcome patterns that we identified. Fourth, we performed multinomial logistic regression analyses to determine whether gender, migration background, and socioeconomic level could predict the membership status of the identified latent patterns. Additionally, we conducted multinomial logistic regression analyses to assess the effect of pattern membership on the HCC levels of the adolescents at study wave 4. We utilized SPSS (Version 24; 97) for paired samples t-tests, while we used Mplus version 8.9 (98) for the remaining analyses.

With the seven latent variables, which had three from the hedonic domain and four from the eudaimonic domain, we employed LCA and LTA as person-oriented typological approaches, as described in previous studies (91, 99, 100). Through this person-oriented approach, we aimed to estimate and gain insight into the continuity of navigation and negotiation levels among students over time. We conducted cross-sectional LCA to determine whether these resilience patterns moved in a developmental forward direction with fewer troubles, or in a backward direction with more troubles. To observe these movements in a differentiated form, all four survey waves were analyzed. This methodology enabled us to group subjects into distinct classes based on the indicators included and then to estimate the probability that a given subject belonged to a specific class, using a person-centered method (92). In contrast to approaches that focus on individual variables, these methods enable identifying latent patterns and trajectories. We determined the most suitable number of patterns through an iterative process and assigned individuals to these patterns based on their posterior probabilities of pattern membership. To handle missing data, we utilized the maximum likelihood method.

LTA is a statistical tool that models transitions in youth violence resilience over time and we chose it for its suitability in meeting this requirement. Upon confirming the optimal number of classes to be four at each time point (performed in the second step of analysis), we conducted an LTA to determine the probabilities of transitions in patterns of violence-resilience outcomes over time, from one latent class to the same class or to another. This procedure estimates the continuity of resilience outcomes at the four adjacent time points. Stability or change is represented by the probability of transitioning to a latent state of a violence-resilience outcome, a pattern that has been recognized, from one study wave to the next.

Information criteria, including the Akaike information criterion (AIC), Bayesian information criterion (BIC), and in particular, the sample-adjusted BIC (SABIC) (91, 101), were employed to guide our determination of the optimal number of patterns. We conducted multiple rounds of LCA and LTA to arrive at the final number of profiles. The level of entropy was considered an indicator of the reliability of the estimate, with values around 0.7 generally deemed acceptable (101–104). For LCA, we applied additional model-fitting criteria, such as the Vuong–Lo–Mendell–Rubin likelihood ratio test (LMR-LRT), the Lo–Mendell–Rubin adjusted likelihood ratio test (aLMR-LRT) (105), and the bootstrapped likelihood ratio test (BLRT). Significant *p* values from these tests indicated improvement over the model with k−1 classes (103. Ultimately, we selected the final model based on a combination of statistical indicators, theoretical considerations, and model simplicity. To mitigate the risk of local solutions, we increased the number of random starts to 1,000 and final optimizations to 100 (106).

## Results

### Analytic step one: differences between all measures across the four study waves

Following our analysis plan, We ran paired t-tests (see **Table 2**) between each pair of adjacent study waves. For self-esteem, we identified a significant increase between study wave 1 and study wave 2 and between study wave 3 and study wave 4. We detected an increase for depression/anxiety between study wave 1 and study wave 2 and between study wave 2 and study wave 3, but a decrease between study waves 3 and 4. For dissociation, we identified only a significant increase when comparing study wave 2 to study wave 3. For self-efficacy, we detected a significant increase only for the comparison between study wave 3 and study wave 4. The levels of undisciplined behavior increased when comparing the respective study waves with study wave 2 having higher levels than study wave 1, study wave 3 having higher levels than study wave 2, and last, study wave 4 having significantly higher levels than study wave 3. We noted highly significant differences for aggression against peers, with increases when comparing study wave 1 to study wave 2 and study wave 2 to study wave 3. Grades in mathematics increased when comparing study wave 3 to study wave 4; between the other study waves, no significant comparisons were displayed.

**Table 2:** Mean Levels (and Standard Deviations) of all LCA/LTA indicators for all waves for the physical abuse subsamples.

Variables	Wave 1 *n* = 509	Wave 2 *n* = 506	Wave 2 *n* = 506	Wave 3 *n* = 561	Wave 3 *n* = 561	Wave 4 *n* = 560
	*M* (*SD*)	*M* (*SD*)	*M* (*SD*)	*M* (*SD*)	*M* (*SD*)	*M* (*SD*)
Self-Esteem	2.54 (.71)	2.81 (.81)***	2.81 (.81)	2.83 (.76)	2.83 (.76)	2.92 (.77)*
Depression/ Anxiety	2.02 (.69)	2.08 (.75)*	2.08 (.75)	2.20 (.80)***	2.20 (.80)	2.08 (.81)***
Dissociation	1.52 (.67)	1.59 (.75)	1.59 (.75)	1.68 (.85)**	1.68 (.85)	1.60 (.80)
Self-Efficacy	2.65 (.68)	2.65 (.69)	2.65 (.69)	2.67 (.70)	2.67 (.70)	2.79 (.68)**
Undisciplined Behavior	1.81 (.54)	1.91 (.62)***	1.91 (.62)	2.05 (.68)***	2.05 (.68)	2.16 (.74)***
Aggression against Peers	1.50 (.58)	1.61 (.64)***	1.61 (.64)	1.77 (.78)***	1.77 (.78)	1.75 (.82)
Grade in Mathematics	4.56 (.72)	4.57 (.72)	4.57 (.72)	4.53 (.75)	4.53 (.75)	4.65 (.82)**

*** = p <.001, ** = p <.01, * = p <.05. Paired samples t-test to the respective next wave.

### Analytic step two: identifying resilience-outcome patterns via LCA

We tested for resilience-outcome patterns via computing a separate LCA for each of the four study waves (study wave 1 *n* = 509; study wave 2 *n* = 506; study wave 3 *n* = 561; study wave 4 *n* = 560).

Among the statistical indicators, we placed primary emphasis on the sample-adjusted Bayesian information criterion (aBIC), which with a lower value indicating a more appropriate fit (91, 101). Following this procedure, we especially focused for each study wave on the class that k−1 had a higher aBIC than k+1 did.

**Figure 1:**
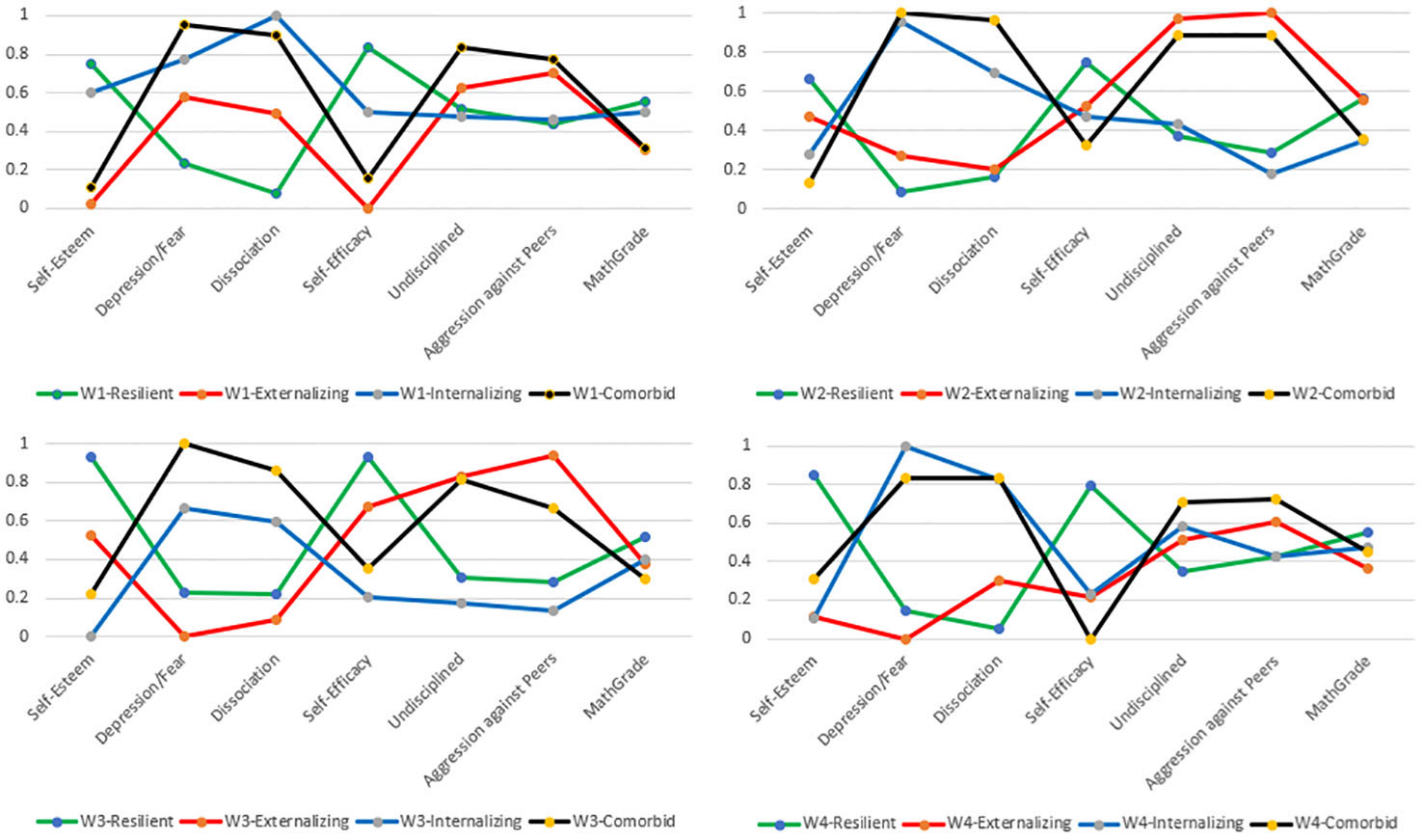
Item response probabilities on the four identified violence resilience-outcome patterns for all four waves.

The aBIC scores dropped between the three- and four-class solutions for all study waves (see **Tables 3**–**6**) more than between the four- and five-class solution, indicating that the four-classes solution is the most appropriate. The VLMR, aLMR, and the BLRT were not conclusive. Additionally, when choosing the four-class solution over the three, we identified a new class being theoretically important, the class we called “internalizing.” By also testing the five-class solution, we did not have an additional theoretical gain. Therefore, we selected a four-class solution for all four study waves (see **Figure 1**).

**Table 3:** Model fit indices for latent class analysis of wave 1.

	Model Fit Criteria					
Models	AIC	BIC	ABIC	ABIC-Drop k-1	Classification Accuracy	BLRT *p*-value
2 Class	4067	4131	4083		.89-.88	<.001
3 Class	4027	4124	4051	32	.73-92	<.001
**4 Class**	**4006**	**4137**	**4038**	**13**	**.74-.92**	**<.001**
5 Class	3992	4157	4033	5	.73-.88	<.001
6 Class	3992	4191	4042	-9	.66-.87	>.05
	Diagnostic Criteria					
	Smallest Class Count (*n*)		Smallest Class Size (%)		Entropy	ALMR LR Test, *p*-value
2 Class	214		42.0		.63	<.001
3 Class	81		15.9		.68	<.01
4 Class	83		16.3		.68	>.05
5 Class	51		10.0		.73	>.05
6 Class	36		7.0		.73	>.05

n = 509. The selected solution is in bold. AIC, Akaike information criterion; BIC, Bayesian information criterion; ABIC, Sample-size adjusted BIC; BLRT, Bootstrap likelihood ratio test; ALMR LR, Lo-Mendell-Rubin Adjusted LRT Test.

**Table 4:** Model fit indices for latent class analysis of wave 2.

	Model Fit Criteria					
Models	AIC	BIC	ABIC	ABIC-Drop k-1	Classification Accuracy	BLRT *p*-value
2 Class	4053	4116	4069		.89-.88	<.001
3 Class	4010	4108	4035	34	.73-92	<.001
**4 Class**	**3994**	**4125**	**4027**	**8**	**.74-.92**	**<.001**
5 Class	3984	4149	4025	2	.73-.88	<.001
6 Class	3985	4184	4035	-10	.66-.87	>.05
	Diagnostic Criteria					
	Smallest Class Count (*n*)		Smallest Class Size (%)		Entropy	ALMR LR Test, *p*-value
2 Class	212		41.9		.70	<.001
3 Class	107		21.1		.75	<.05
4 Class	99		19.5		.68	>.05
5 Class	62		12.2		.72	<.05
6 Class	33		6.5		.72	>.05

n = 506. The selected solution is in bold. AIC, Akaike information criterion; BIC, Bayesian information criterion; ABIC, Sample-size adjusted BIC; BLRT, Bootstrap likelihood ratio test; ALMR LR, Lo-Mendell-Rubin Adjusted LRT Test.

**Table 5:** Model fit indices for latent class analysis of wave 3.

	Model Fit Criteria					
Models	AIC	BIC	ABIC	ABIC-Drop k-1	Classification Accuracy	BLRT *p*-value
2 Class	4649	4714	4667		.92-.94	<.001
3 Class	4590	4689	4616	51	.78-.92	<.001
**4 Class**	**4555**	**4689**	**4591**	**25**	**.81-.90**	**<.001**
5 Class	4544	4713	4589	2	.84-.89	<.05
6 Class	4546	4749	4600	-11	.76-.91	>.05
	Diagnostic Criteria					
	Smallest Class Count (*n*)		Smallest Class Size (%)		Entropy	ALMR LR Test, *p*-value
2 Class	179		31.9		.76	<.001
3 Class	118		21.0		.72	>.05
4 Class	76		13.5		.77	<.01
5 Class	46		8.2		.79	<.05
6 Class	38		6.8		.76	>.05

n = 561. The selected solution is in bold. AIC, Akaike information criterion; BIC, Bayesian information criterion; ABIC, Sample-size adjusted BIC; BLRT, Bootstrap likelihood ratio test; ALMR LR, Lo-Mendell-Rubin Adjusted LRT Test.

**Table 6:** Model fit indices for latent class analysis of wave 4.

	Model Fit Criteria					
Models	AIC	BIC	ABIC	ABIC-Drop k-1	Classification Accuracy	BLRT *p*-value
2 Class	3983	4048	4000		.83-.92	<.001
3 Class	3959	4059	3986	14	.73-.80	<.001
**4 Class**	**3948**	**4082**	**3983**	**3**	**.74-.85**	**<.001**
5 Class	3940	4108	3985	-2	.65-.96	<.05
6 Class	3937	4141	3992	-7	.67-.84	>.05
	Diagnostic Criteria					
	Smallest Class Count (*n*)		Smallest Class Size (%)		Entropy	ALMR LR Test, *p*-value
2 Class	169		30.2		.58	<.001
3 Class	76		13.6		.58	>.05
4 Class	43		7.7		.62	>.05
5 Class	39		7.0		.61	<.05
6 Class	27		4.8		.67	>.05

n = 560. The selected solution is in bold. AIC, Akaike information criterion; BIC, Bayesian information criterion; ABIC, Sample-size adjusted BIC; BLRT, Bootstrap likelihood ratio test; ALMR LR, Lo-Mendell-Rubin Adjusted LRT Test.

We detected a (a) class called “resilient” with higher levels of self-esteem, self-efficacy, and math grade, and lower levels on depression/anxiety, dissociation, aggression against peers; (b) class called “externalizing” with lower levels on five indicators but higher levels on aggression against peers and discipline problems at school; (c) class called “internalizing” with lower levels on five indicators but higher levels on depression/anxiety and dissociation; and (d) a class called “comorbid” with lower levels on self-esteem, self-efficacy, and math grade but higher levels on aggression against peers, discipline problems at school, depression/anxiety, and dissociation.

### Analytic step three: LTA to indicate significant differences in the longitudinal classification variables on the identified resilience-outcome patterns

The third step involved using LTA to identify significant variations in longitudinal classification variables among the identified cross-sectional patterns. The same three hedonic and four eudaimonic classification variables used in the LCAs were used for the LTA as well, with **Table 7** providing model fits. We conducted the LTA with two to six latent classes to verify if the conditional response probabilities were time-invariant.

**Table 7:** Latent transition analysis model fit statistics to select longitudinally the number of resilience status classes over four waves.

Classes	AIC	aBIC	Entropy	Samples	Classification Accuracy
**2**	16421	16445	.71	w1: 220/343; w2: 239/324; w3: 214/349; w4: 238/325	.88-.89
**3**	16180	16227	.68	w1: 249/191/123; w2: 243/208/112; w3: 227/183/153; w4: 211/179/173	.73-.89
**4**	**16013**	**16091**	**.69**	**w1: 102/103/116/242; w2: 140/84/101/238; w3: 113/77/160/213; w4: 81/106/180/196**	**.66-.89**
**5**	15914	16029	.81	w1: 60/189/93/126/95; w2: 58/189/73/103/140; w3: 52/165/77/165/104; w4: 73/69/163/141/117	.60-.84
**6**	15859	16018	.73	w1: 50/84/68/123/163/69; w2 45/75/64/91/177/111; w3 49/77/56/157/143/81; w4 50/87/76/232/54/64	.32-.78

AIC, Akaike information criterion; aBIC, adjusted Bayesian information criterion; the chosen solution is highlighted in bold.

Between the three- and four-class solution, we noticed the highest aBIC drop (−Δ136). Corresponding to this, the aBIC difference between the four- and five-class solution was far lower (−Δ 62) and indicated the expected elbow profile and suggested a four-class solution as appropriate. Because of the rule favoring more rigorous models (rule of deference), a four-class solution was chosen for the longitudinal analyses using LTA.

We had the additional possibility (see **Table 8**) to check for changes of the respective students between the identified four classes. The class we called “resilient” (study wave 1 = 18.3%; study wave 2 = 14.9%; study wave 3 = 13.7%; study wave 4 = 18.8%) showed a high stability over four study waves with a slight drop at study waves 2 and 3. The class we called “externalizing” (study wave 1 = 18.1%; study wave 2 = 24.9%; study wave 3 = 20.1%; study wave 4 = 14.4%) also showed high stability but it was less pronounced at study wave 4 in comparison to study wave 1, contrasting with the class we called “internalizing” (study wave 1 = 20.6%; study wave 2 = 17.9%; study wave 3 = 28.4%; study wave 4 = 32.0%), which had an enormous increase. Last, combined with the slight decrease of the externalizing class, we had a decrease for the class called “comorbid” (study wave 1 = 43.0%; study wave 2 = 42.3%; study wave 3 = 37.8%; study wave 4 = 34.8%).

**Table 8:** Estimated longitudinal probabilities by LTA over four waves of the four resilience patterns.

	Wave 1	Wave 2	Wave 3	Wave 4
Resilient	n=103; 18.3%	n=84; 14.9%	n=77; 13.7%	n=106; 18.8%
Externalizing	n=102; 18.1%	n=140; 24.9%	n=113; 20.1%	n=81; 14.4%
Internalizing	n=116; 20.6%	n=101; 17.9%	n=160; 28.4%	n=180; 32.0%
Comorbid	n=242; 43.0%	n=238; 42.3%	n=213; 37.8%	n=196; 34.8%

### *Analytic step four: testing s*ociodemographic *factors predicting the latent pattern membership at study wave 4*

After identifying the classes for all four study waves, we tested for study wave 4 sociodemographic factors predicting the latent pattern membership using a multinomial logistic regression (see **Table 9**): Gender, migration background, and socioeconomic status were included as sociodemographic predictors to determine which individual characteristics increased the likelihood of being a member in the comorbid pattern, as the pattern with the most vulnerable adolescents.

**Table 9:** Multinomial logistic regression of sociodemographic covariates in the four patterns, for LTA wave 4.

LCA Wave 4 Class	Predictor	B	*SE*	Wald statistic	*p*	*OR*	Prediction in % Pseudo-R^2^		
							Cox & Snell	Nagelkerke	Mac-Fadden
resilient	Intercept	-.03	.18	.03	.855		11.5	12.4	4.5
	Gender (1 female; 2 male)	-1.21	.28	18.64	<.001	.30			
externalizing	Intercept	-.05	.19	.08	.780				
	Gender (1 female; 2 male)	-1.78	.33	28.36	<.001	.17			
internalizing	Intercept	-.29	.20	2.17	.141				
	Gender (1 female; 2 male)	.07	.25	.07	.789	1.07			
resilient	Intercept	-.57	.16	13.35	<.001		1.9	2.1	.7
	Migration background (0 no MB, 1 with MB)	.11	.29	.14	.706	1.12			
externalizing	Intercept	-1.05	.19	32.19	<.001				
	Migration background (0 no MB, 1 with MB)	.76	.30	6.47	.011	2.14			
internalizing	Intercept	-.23	.14	2.62	.106				
	Migration background (0 no MB, 1 with MB)	-.097	.27	.12	.723	.91			
resilient	Intercept	-.36	.19	3.73	.054		.4	.5	.2
	Socio-Economic Status (0 high, 1 low)	-.35	.27	1.77	.184	.70			
externalizing	Intercept	-.68	.21	10.70	.001				
	Socio-Economic Status (0 high, 1 low)	-.19	.29	.45	.504	.82			
internalizing	Intercept	-.14	.18	.63	.428				
	Socio-Economic Status (0 high, 1 low)	-.22	.24	.82	.366	.80			

SE, Standard Error; OR, Odds Ratio. Reference LCA wave 4 pattern is “comorbid” with high levels on both externalizing and internalizing symptoms.

Gender was strongly predictive to class membership at study wave 4. The prediction strength for male students was more than three times lower as for female students for being in the class “resilient” compared to the class “comorbid”. The probability of male students being in the class “externalizing” compared to the class “comorbid” was about six times lower than for female students. No gender differences between the classes “comorbid” and “internalizing” were displayed. The prediction strength of migration background was only significant for the comparison between the “externalizing” and “comorbid” classes with students with a migration background having a more than twice as high a probability of being in the “externalizing” than in the “comorbid” class. We detected no significant prediction when analyzing the connection between SES and the latent pattern membership at study wave 4.

### Analytic step five: on the understanding of the longitudinal classification changes on the identified resilience-outcome patterns between study wave 3 and study wave 4

#### The HCC-*subsample at* study wave 4

To test whether the developmental changes in adolescents who had experienced violence were reflected in the biomarkers, we took hair samples from the overall sample. Regarding participation from the overall sample at study wave 4 (*N* = 1,609) compared to those in the HCC study (*n* = 229), we checked for differences of the tested sociodemographic variables. Approximately every 7th student (14.23%) participated in the HCC sampling. Participants did not differ significantly in gender (*t*[2092] = −1.192, *p* = .235), SES (*t*[1762] = −1.826, *p* = .069), or prevalence of physical abuse by parents (*t*[2119] = 1.442, *p* = .150). We identified only for migration background a significant difference (*t*[2095] = 1.970, *p* = .049), with the HCC sample having a slightly higher proportion of students with a migration background than the overall sample (47% in comparison to 54%). Due to this, the two samples were considered comparable. Just 1 year after COVID-19 and considering the difficulties of parents consenting during the pandemic, we consider this a positive result because parents and students had to consent a second time and that was specific to the HCC sampling.

#### On the relation between HCC level and pattern membership at study wave 4

We introduced the HCC measures to identify the connection between HCC levels and the respective resilience classes. Due to this, we assume that the HCC level affects the allocation probability to the respective classes. Within the HCC subsample of *n* = 229, we had *n* = 68 students being physically abused by their parents with *n* = 14 (20.65%) being assigned to the resilient pattern, *n* = 16 (23.55%) in the externalizing pattern, *n* = 17 (25.0%) in the internalizing, and *n* = 21 (30.9%) being at the comorbid pattern. When comparing the distribution of the HCC subsample with the subsample of all physically abused adolescents *(n = 563)*, we found compelling differences: We first checked by multinomial regression analysis for possible differences on the HCC levels between the four identified classes at study wave 4. The multinomial logistic regressions for HCC levels were calculated with the class “comorbid” as the reference predictor for the probability of reporting. The overall prediction of the subsample is 17.9% Nagelkerke (Chi^2^ (3, *N* = 68) = 12.437, *p* <.01). Considering that we used only one predictor, the HCC level, we believe that we have established the reliability of our results. We also found that in our HCC subsample, adolescents with higher levels on HCC had a more than four times higher (OR = 4.25) probability of being assigned to the comorbid group than to the resilient group (Wald-test (1, *N* = 68) = 3.521, *p* <.05) and the probability of being in the comorbid group is almost 13 times (OR = 12.75) higher compared to adolescents of the group externalizing (Wald-test (1, *N* = 68) = 10.091, *p* <.001.). No differences on the HCC levels were found when comparing the comorbid group to the internalizing group (Wald-test (1, *N* = 68) = 2.155, *p* >.05). Interestingly, adolescents with “just” internalizing symptoms had similar HCC levels to adolescents of the comorbid class, indicating the specific relation between internalizing symptoms and higher HCC levels. Considering that the comorbid class at study wave 4 is the biggest class (about 35%), a very high proportion of the adolescents suffer from high HCC levels. We identified, especially for the comorbid group, high HCC levels and significantly lower levels for the resilient and the externalizing group (see **Table 10**).

**Table 10:** Mean Levels and Standard Deviations of hair cortisol concentrations for the four patterns (n = 68 abused adolescents).

Patterns	Sample size	*M* in pg/mg	*SD* in pg/mg
Resilient	14	5.95	3.21
Externalizing	16	5.50	2.11
Internalizing	17	9.34	13.24
Comorbid	21	8.16	4.82

Additionally, the HCC levels of physically abused adolescents can not only be viewed in the dichotomy of resilient and nonresilient but must also be specified if the adolescents have internalizing, externalizing, or comorbid symptoms who seem to be far closer connected to the higher HCC levels than adolescents with “just” externalizing symptoms. We checked for this specific assumption by computing a new variable having two categories, “1”, resilient adolescents, with the adolescents of the resilient class (*n* = 14), and “2”, nonresilient adolescents, (*n* = 54) with adolescents of the other three classes (*n* = 16 externalizing; *n* = 17 internalizing; *n* = 21 comorbid). We then ran a t-test to analyze for possible mean differences of HCC levels on the two categories, and we identified no significant results: The mean HCC-mean score of the resilient adolescents (*M* = 5.95 pg/mg; *SD =* 3.21) was not significantly lower than the mean-HCC level (*M* = 7.74 pg/mg; *SD =* 8.08) of the nonresilient adolescents (*t*(66) = .81, *p* = .419).

We checked also for the similar relations by analyzing the HCC mean differences between the nonabused adolescents (*n* = 153) and the abused adolescents by probing two solutions: First the overall HCC-levels comparison between nonabused adolescents (*n* = 153) and abused (*n* = 68) adolescents by a t-test and second, by nonabused adolescents to the four specific abused groups (resilient *n* = 14, externalizing *n* = 16, internalizing *n* = 17, and comorbid *n* = 21) by a multinomial regression. On the first assumption, that there is an overall difference of the mean-HCC levels between the abused (*M* = 7.37 pg/mg; *SD =* 7.36) and the nonabused (*M* = 6.54 pg/mg; *SD =* 4.76) adolescents, we identified no significant results (*t*(217) = −1.00, *p* = .316). Testing the second assumption, we noted that the overall prediction of the HCC subsample (*n* = 229) is 7.0% Nagelkerke (Chi^2^ (4, *N* = 229) = 13.912, *p* <.01). We identified there was only a significant HCC-level difference when comparing the nonabused adolescents to the comorbid group, with nonabused adolescents having an almost five times (OR = .20) lower probability having higher HCC levels than the adolescents from the comorbid group (Wald-test (1, *N* = 229) = 7.31, *p* <.01), but not to the other three abused adolescents’ groups (resilient group Wald-test (1, *N* = 229) = .046, *p* >.05; externalizing group Wald-test (1, *N* = 229) = 2.66, *p* >.05; internalizing group Wald-test (1, *N* = 229) = .841, *p* >.05).

## Discussion

We examined the combined impact of domain-specific hedonic and eudemonic factors in relation to long-term resilience, despite physical abuse at home, and additionally associated physical abuse in the family with blunted cortisol reactivity (26, 27) and HPA as a brain response with an increased release of stress hormones (78). Overall, our results highlight the importance of hormones for understanding resilience status in adolescence. Cortisol is increasingly released in response to a stressor (cortisol reactivity; 26). Through the use of a person-centered analytical approach utilizing latent class and transition analysis, we were able to identify and compare four distinct patterns of violence-resilience over time. Our research confirms that violence-resilience is a multifaceted concept, encompassing both hedonic and eudaimonic indicators of protection and risk. Rather than solely focusing on the absence of psychopathology, our analysis included factors related to both emotional well-being (such as high self-esteem and low levels of depression/anxiety) and personal growth such as self-efficacy, along with low levels of aggression toward others). Our findings also showed a significant relationship between elevated cortisol levels and a pattern of comorbid internalizing and externalizing symptoms, supporting the notion of lasting changes in brain structure.

Just imagine having a shortsighted adolescent student, but instead of giving them glasses or offering them a seat at the front of the classroom, we demand they should do better, push themselves to “just do it,” and read the content of the board from wherever they are seated at school. Even if this example sounds not just undesirable but also cruel, we are practically doing the same thing with physically abused adolescent students who exhibit a symptomatic pattern. We ask them to behave, relate, and develop emotionally and academically in the expected way, even while their behavior and their brain functions have been adversely affected by abuse-related higher cortisol levels that biologically hinder their functioning and development.

Family is considered a safe and fostering space for adolescents, but globally, about 25% of adolescents experience severe forms (e.g., blows, kicks) of physical abuse by their parents (15, 23), which may lead to long-lasting alterations in adolescents’ brains (27). Meaning, when looking at a school class of about 20 students coming to school on a given day, about four students have experienced physical abuse at home. How should these students perform, behave, and feel at school? How should they connect to other classmates as well to their teachers with their stress system constantly highly elevated?

### Hedonic and eudaimonic patterns of violence-resilience

Resilience research has continually evolved over the years, with a commitment to yielding meaningful outcomes and a willingness to embrace innovative approaches that offer diverse perspectives. It has gone through four distinct methodological and thematic waves, each challenging existing concepts and frameworks and that lead to the development of further advanced and comprehensive methodologies (35). Resilience research also focuses on the system’s domain-specific adaptation, the capacity of the respective dynamic systems to withstand or recover from disturbances (28). Especially, when studying positive youth development within family, we have to pose the question if families are doing what they should to support students and if this familial system develops in conjunction with adolescent student needs (61) to foster their neurodevelopment and ultimately, not to induce long-lasting alterations in brain structures involved in depression, anxiety disorders, dissociation, and aggression such as the amygdala, the hippocampus, and the prefrontal cortex (9). What insights can we gain into the (in-)stability of resilience?

Establishing the resilience pathways longitudinally is particularly important in enhancing the understanding and definition of this phenomenon. While we currently lack information regarding the persistence of violence-resilience outcomes, encompassing both hedonic and eudaimonic aspects, it is essential to explore what transpires after initially detecting resilience at study wave 1. In particular, it is crucial to investigate whether the resilience patterns identified at study wave 1 lead to different resilience trajectories. As a first result, we confirmed previous studies (15) and our first hypothesis, that we could also identify four classes over a period of three years. Following Aksoy et al. (30) and confirming our second and partly third hypothesis, the class we called “resilient” showed high stability over four study waves with a slight decline in study waves 2 and 3. Interestingly, the indicator levels of the resilient classes were particularly high in study waves 2 and 3, which would also explain the decline in the proportions. At the same time, study waves 2 and 3 were a period of uncertainty due to COVID-19, which also led to an increased use of mental health care among children and adolescents (107). However, contrary to COVID-19 research on student achievement (108), the resilient class showed no differences in mathematics scores compared to the other classes, thus indicating relative stability in academic performance, but this is only true for the resilient class. Therefore, resilience development seems to buffer academic performance problems when encountering acute adversity in our sample. The class we termed “externalizing” also showed high stability, increasing proportionally over time, but was less pronounced in study wave 4 compared to study wave 1. This could be potentially because the externalizing class still had stronger comorbid symptom development in the first study wave and that these symptoms clearly developed into externalizing ones by the next three measurement time points. Therefore, it is possible to assume that the adolescents possibly fluctuated between the externalizing and the comorbid class at the second and third measurement time points. This contrasts with what we call the internalizing class, which shows a big proportional increase over time. Looking at this class’s development, it fluctuated across the three measurement points, with self-esteem in particular suffering greatly and only recovering very slightly in the fourth study wave. Finally, we recorded a decrease in the “comorbid” class, which is a very pleasing result from our perspective because this class captures the most vulnerable combination of developmental impairments (109).

Resilience research has examined sociodemographic factors such as gender, migration background, and SES, but their effects on resilience outcomes have yielded inconclusive results. Thus, our fourth research hypothesis related to pattern membership in terms of individual sociodemographic characteristics. We inquired which characteristics are represented in the resilient pattern and are thus protective factors for belonging in the resilient pattern at study wave 4. We assumed that pattern membership would differ by gender (39, 40) and SES (110). Therefore, we performed multinomial logistic regression with the sociodemographic variables. Results showed, that female gender was a protective factor for being in the resilient pattern because female students had a three times higher likelihood than male students to be in the resilient pattern. In contrast, male students were more likely to be represented in the externalizing pattern. These findings are consistent with existing results (15). Participants with a migration background were more likely to be in the comorbid pattern than in the externalizing pattern, thus struggling with internalizing as well as externalizing behaviors. This is consistent with previous findings from Favre et al. (40), who showed that immigrant youth were more likely to belong to a pattern that included both externalizing and internalizing behavior problems. Contrary to what was assumed, SES did not have predictive significance. Thus, the fourth hypothesis must be partially rejected.

### Linking resilience to violence and biomarkers

The present study aimed to expand the understanding of the relationship between hormones and socioemotional resilience in adolescence despite physical abuse in the family. As it becomes not only feasible but also thinkable to measure biological markers such as genes or hormones and to combine these insights with psychological markers that questionnaire data yield, we have to answer questions on adaptive functioning of complex systems differently because the roles of the individual students’ respective psychobiological systems interact with the psychobiological systems of other students and teachers in the classroom. These interactions influence adaptive patterns and the interplay of schools’ functioning with students’ development and may be identified by complex statistical tools (64). However, the system is subject to developmental changes, making adolescence and puberty a sensitive time slot for programming the response to stress (111). Recent research also shows the effects of stress on the microbiome and establishes a link with diet (112), which could be influenced by providing specific foods for the students concerned. The gut microbiota produce many hormones for general well-being, such as serotonin and dopamine. A negative influence from physical and emotional stressors can affect this metabolism and lead to a change in the production of these hormones. If the spiral continues, this cycle can even lead to anxiety in the long term (113).

Campbell and Ehlert (114) note in their systematic review that stress reactivity, “is a complex phenomenon involving several response systems, namely cognitive, emotional, physiological and behavioral responses” (p. 1130). We have generated proof that speaks in favor of this statement by identifying especially the comorbid resilience pattern to connect to higher levels on HCC. Thus, in relation to physical abuse as an acute stressor, different response systems need to be considered more closely. As Fogelman and Canli (115) pointed out, cortisol awakening responses are particularly amplified in early life stress events experienced in the forms of sexual, physical, or emotional abuse compared to other forms of early life stress and it may affect especially amygdala, the hippocampus, and the prefrontal cortex (9). Interestingly, our data show that this holds for the symptomatic patterns but not for the resilience pattern that consists of almost every fifth adolescent having experienced physical abuse at home by their parents. Therefore, we can assume that violence resilience not only protects adolescents from psychiatric problems but also on a physiological level.

We have also confirmed the main result of Roydeva and Reinders (7) systematic review that cortisol levels highly relate to dissociative pathology, and further gained the insight that dissociative symptoms are a psychosocial stress response. Additionally, Simeon et al. (116) found in their study with individuals with borderline personality disorder that those individuals with high dissociation levels also showed the significantly strongest cortisol stress reactivity compared to individuals with low or absent dissociation levels. Considering these results on the psychobiological state of abused adolescents, adolescent students who have suffered abuse may respond to the learning expectations of schooling with agency and self-determination which then tends to increase their self-esteem and makes it less likely they will suffer from depression. Alternatively, there may be a mismatch between their own expectations and those of schooling, such that the pressure of schooling adds to their sense of futility and increases the likelihood they will suffer from depression. Our study suggests that the proportion of students who thrive despite abuse is fairly large, and it suggests that schools should avoid the implicit and explicit messages to the remainder of students that they cannot learn, for whatever reason. Especially, whole school approaches on supporting resilience processes when students are experiencing mental health problems such as depression/anxiety, dissociation, and aggression are requested (117). Globally validated results on prevention and intervention on whole school approaches show clear evidence of this approach (118). To foster the respective adolescents’ development, we also have to consider supporting teachers and school principals to identify adolescents’ developmental symptoms (119). The explicit mechanism of abuse’s effects on children are still opaque, and some youth manage to avoid the harms to their learning capacity without much help, but others need extra assistance. Reading interventions are one example where there is a choice between acceptance of differences in abilities or research-based interventions that intervene early to prevent the cognitive and socioemotional blockages that sometimes result from abuse (e.g., 120).

Additionally, and to mitigate the actual risk on physical abuse at home, we have to question what it means to the so-called modern and civilized societies that families are a supposed “heaven,” but for every fourth adolescent, they are experienced more as a “hell” on earth. Our study complements the topic of NDDs and resilience in families by looking at the perspective of physical abuse as a possible cause of NDDs as well as how adolescents navigate it. It cannot be ruled out that this creates a vicious circle if NDDs are triggered by parents and in turn, put a strain on the parents or the entire family. This makes it all the more important to understand resilience patterns to help adolescents get out of this cycle.

## Limitations

The sample drawn was a true reflection of Switzerland only. Hence, it is imperative to exercise restraint while interpreting the results and refrain from extrapolating the same to other countries. Conducting international research on the subject is warranted.

In this study, we have thus far been able to address the question of how we could support adolescent students suffering because of their family abuse experiences so they perform and feel better. How could we support these adolescents to reduce the HCC levels? Might the only way out be to learn to deal with that, or can we really make a real contribution? Furthermore, with this approach we are supporting these adolescents in different ways than by just requesting that they take a pill.

A further limitation is that the interaction of HCC with testosterone could not be included in this study. Grotzinger et al. (121) found that a lower cortisol level correlates with a higher level of aggression only when testosterone levels were high. Whereas Platje et al. (122) found that low cortisol levels correlate with higher levels of aggression only when testosterone levels were high, referring to the dual-hormone hypothesis (74).

White et al. (24) found that abused children initially exhibited hypercortisolism in early childhood, which transitioned to hypocortisolism in adolescence. Elevated HCC levels predicted internalizing symptoms, while reduced HCC levels related to externalizing symptoms in middle childhood and adolescence. The authors suggested that HCC could mediate the effects of maltreatment on externalizing symptoms, although longitudinal studies are needed to verify this. A comprehensive longitudinal study, besides hair analysis, could be conducted on a group of individuals at fixed times of the day to exclude cardiac rhythms, using saliva or dried blood samples to test for these effects.

Thus far, we did not analyze for second-order effects, such as classroom composition, on an individual’s resilience process. As we know from previous studies, not only the link to the other students’ mental health level in the classroom exists but also to the well-being of the respective teachers (123).

Even if the tested HCC subsample did not differ significantly in its composition when compared to the overall sample, we must consider that approximately only every 10th student participated in the HCC sampling. Due to this, we have to be careful when extrapolating the HCC results.

Due to the sample size, we did not examine the severity or frequency of physical abuse in our analyses. We expect that a higher range of severity or frequency may have had an impact on membership in the respective resilience patterns, here specifically the comorbid pattern, and on transitions from one pattern to another over time. It is important to recognize that our findings may have been affected by the COVID-19 pandemic, especially because study wave 1 and study wave 2 were gathered earlier in the pandemic, and study wave 3 and study wave 4 occurred after the pandemic’s end. Previous research has shown that the pandemic’s initial stages had adverse effects on mental well-being and self-determination, which could have influenced our results (124, 125).

## Data availability statement

The raw data supporting the conclusions of this article will be made available by the authors, without undue reservation.

## Ethics statement

The research project received authorization by the ethics committee of the School of Education, University of Applied Sciences and Arts Northwestern Switzerland. The studies were conducted in accordance with the local legislation and institutional requirements.

## Author contributions

WK: Conceptualization, Data curation, Formal analysis, Funding acquisition, Investigation, Methodology, Validation, Writing – original draft, Writing – review & editing. DA: Investigation, Methodology, Writing – original draft, Writing – review & editing. CF: Investigation, Methodology, Writing – original draft, Writing – review & editing. JA: Writing – original draft, Writing – review & editing. SG: Writing – original draft, Writing – review & editing. KG: Formal analysis, Methodology, Writing – original draft, Writing – review & editing. SA: Writing – original draft, Writing – review & editing. DM: Methodology, Visualization, Writing – original draft, Writing – review & editing.
